# Characterizing thermal-oxidation behaviors of nuclear graphite by combining O_2_ supply and micro surface area of graphite

**DOI:** 10.1038/s41598-018-31493-4

**Published:** 2018-09-07

**Authors:** Yangping Zhou, Yujie Dong, Huaqiang Yin, Zhengcao Li, Rui Yan, Dianbin Li, Zhengwei Gu, Ximing Sun, Lei Shi, Zuoyi Zhang

**Affiliations:** 1 Institute of Nuclear and New Energy Technology, Collaborative Innovation Center of Advanced Nuclear Energy Technology, Key Laboratory of Advanced Reactor Engineering and Safety of Ministry of Education, Tsinghua University, Beijing, 100084 China; 2 State Key Laboratory of New Ceramics and Fine Processing, Key Laboratory of Advanced Materials (MOE), School of Materials Science and Engineering, Tsinghua University, Beijing, 100084 China

## Abstract

The effects of different parameters on oxidation rate are non-linear, interactive and diversified in which the change of adequacy of O_2_ supply is an important indicator. The influence of microstructure on oxidation rate became stronger worsening the fitting linearity to calculate the activation energy based on present method with the decreased adequacy of O_2_ supply due to the increase of temperature, the decrease of gas flow rate, etc. Here, we proposed a method to characterize thermal-oxidation behaviors of nuclear graphite by combining O_2_ supply and micro surface area of graphite. The proposed method improved the linearity and reduced the standard error of Arrhenius plots of oxidized graphite IG-110 (10 L/min reactant gas) and ET-10 (0.2 L/min reactant gas). The value of activation energy of graphite IG-110 oxidized under ASTM D7542 condition is calculated as 220 kJ/mol by this method echoing the results of previous studies with sufficient O_2_ supply. For the conditions with less O_2_ supply at low gas flow rate and/or high temperature, the change of microstructure of oxidized graphite should be obtained as an important factor influencing oxidation rate of graphite.

## Introduction

Nuclear grade graphite, because of its anti-radiation performance and excellent mechanical properties, is widely used in High Temperature Gas-cooled Reactor (HTGR)^1,2^ and molten salt reactor^3,4^ as the material of structure, moderator, reflector and fuel element. In addition, graphite is also applied to electronics, chemical engineering and other fields. When a HTGR runs under normal operation, the impurities are introduced due to graphite degasification and small leakage of H_2_O from the secondary side to the primary side through the heat-exchanger tubes of steam generator^5^, which inevitably corrodes the graphite components at temperature higher than 400 °C^6^. In addition, the oxidation/corrosion of the graphite will be accelerated during an air or water ingress accident^7,8^. It has been found that the mechanical and thermal properties of graphite will be deteriorated by its oxidation/corrosion, shortening the lifetime of the graphite components^9–11^. In addition, the reaction of air and graphite can cause temperature increase by heat generation and accumulation of explosive CO gas in the reactor during an air ingress accident^12^. Despite of the above negative effects, the thermal oxidation can sever as an option to treat a large scale waste of nuclear graphite^13^.

Consequently, the oxidation/corrosion of graphite arises to be a crucial issue to assess the economy and safety of a HTGR among which O_2_ oxidation is usually the fastest reaction. There are two main concerned reactions related with O_2_ oxidation of graphite when temperature is less than 900 °C^14^:

1 $$C-O\,reaction:\,C+(1-\frac{x}{2}){O}_{2}=\mathrm{(1}-x)C{O}_{2}+xCO\,{\rm{\Delta }}H=-393.5(1-x)-\,110.5x\,(kJ/mol)$$

2 $$CO\,combustion:\,CO+\frac{1}{2}{O}_{2}=C{O}_{2}\,\,\,\,\,\,\,\,{\rm{\Delta }}H=-\,283.0\,(kJ/mol)$$

The reaction (1) is considered as the intrinsic oxidation reaction between O_2_ and graphite. Reaction (2) (CO combustion) may influence the reaction (1) (oxidation reaction) by varying O_2_ supply and energy balance of reaction (1). Related studies on nuclear graphite had been widely carried out in investigate the oxidation behaviors of various graphite. Present studies mainly consisted of two categories according to their purposes and reactant gas flow rates, high gas flow rate based on common sense of material engineering and low gas flow rate for accident conditions of reactor usually driven by natural convection. The studies with high gas flow rate usually had the oxidation conditions close to ASTM D7542^15^ (originally approved in 2009) with related sufficient O_2_ supply (e.g. 10 L/min air flow) and a cylinder geometry specimen (e.g. D = H = 25.4 mm) based on the common sense of material engineering. On the contrast, the studies with low gas flow rate usually had the diversified conditions (gas flow rate, O_2_ concentration and geometry of specimen) with related insufficient O_2_ supply according to the accident analysis for different reactors.

Fundamentally, the graphite oxidation rate relates with temperature, difficulty of oxidation (activation energy) and reactant supply including O_2_ concentration, gas flow rate and micro structure and geometry of graphite. The effects of different parameters on oxidation rate are non-linear, interactive and diversified. Present studies mainly focused on the relations between oxidation rate and temperature or O_2_ concentration which discussed the activation energy or reaction order of graphite oxidation respectively. The influences of gas flow rate and microstructure of graphite on oxidation rate are usually ignored.

For oxidation behaviors with high gas flow rate, the microstructure of graphite, such as surface area, was indicated to be a constant object at a certain range of Mass Loss (ML), which was independent from temperature and O_2_ supply^16,17^. Simultaneously, the standard of ASTM D7542 recommended the method to calculate the activation energy of graphite oxidation using average oxidation rates from 5% to 10% ML of specimen. Contescu *et al*. indicated the adequacy of O_2_ supply could be the indicator whether ASTM D7542 is applicable^18^. The adequacy of O_2_ supply indicated by the ratio of O_2_ supply to consumed O_2_ should be around 10 or higher to avoid the departure of the oxidation mechanism from chemical kinetic regime.

However, several studies^12,19–23^ on graphite IG-110 got the quite different values of activation energy, whose conditions were close to that recommended by ASTM D7542. A study indicated that recommend condition by ASTM D7542 can not guarantee sufficient O_2_ supply for oxidation of graphite IG-110, and therefore the increased insufficiency of O_2_ supply resulted in decreased values of activation energy^24^. In addition, a recently study found the nonlinearity of the average reaction rate (from 5% to 10% ML) with the increase of air flow rate^25^ implicating other factors such as microstructure may play an unignorable role in graphite oxidation.

On the other hand, with low gas flow rate, the studies considered the actual accident conditions of reactor usually concerning the oxidation behaviors of diversified geometries of specimens^11,24,26–30^. Some of these studies characterized the oxidation behaviors according to oxidation rate at a same oxidation time^11,24,26,27^ not a same range of ML since the accident analysis mainly concerned the situation based on the time criterion after the air ingress accident. All above studies with much lower supply O_2_ (e.g. 0.2 L/min gas flow) usually got quite lower values of activation energy than those under close conditions with ASTM D7542 (10 L/min gas flow) for same graphite.

Based on the above phenomena, we concluded the adequacy of supply O_2_ may act as an indicator to characterize the oxidation behaviors. The influence of microstructure on oxidation rate became stronger worsening the fitting linearity to calculate the activation energy based on the present method when the adequacy of O_2_ supply decreased due to the increase of temperature, the decrease of gas flow rate, etc. For the situation of a related high gas flow rate (around 10 L/min), the oxidation behaviors should be discussed more in detail in terms of the type of graphite and actual O_2_ supply. Since the conditions recommended by ASTM D7542 were determined by the experiments of graphite NBG-10, PGXW and H4650^31^, the sufficiency of O_2_ supply may be hurt by the increased O_2_ consumption of the oxidation of some other graphite, such as IG-110. For the situation of a low gas flow rate (e.g. 0.2 L/min) concerning the accident condition, the O_2_ supply became insufficient quickly with the increase of temperature, and therefore apparently worsened the linearity of the fitting calculation^24,26^.

All mentioned above call a higher adaptive method to characterize the oxidation behaviors of nuclear graphite in a wide range of reactant supply (with gas flow rates of 10 L/min and 0.2 L/min, cylinder and oblate rectangular specimens, etc.). After re-examining relations among various factors determining the oxidation rate, we proposed a method to characterize the oxidation behaviors regarding concentration of O_2_, gas flow rate and surface area of open pore of graphite. Two typical scenarios were applied to validate the proposed method. The first is the oxidation of graphite IG-110 with high gas flow rate (10 L/min) whose conditions followed ASTM D7542. The second is the oxidation of graphite ET-10 with low gas flow rate (0.2 L/min) where the oblate rectangular specimens were oxidized. For the high gas flow rate (10 L/min), we got the related data including surface areas from previous study^32^. The calculation result of activation energy of graphite IG-110, 220 kJ/mol, echoed the results of previous studies with more adequate O_2_ supply, 218 kJ/mol^12^ and 222 kJ/mol^19^, compared with 201 kJ/mol^21^ or 205 kJ/mol^32^ under the experiment condition recommended by ASTM D7542. For the low gas flow rate (0.2 L/min), nuclear graphite ET-10, produced by IBIDEN Co. Ltd, was oxidized by a 0.2 L/min mixture gas (helium and O_2_, 10% or 20% O_2_ mole fraction) at 650–850 °C. The oxidation facility mainly consisted of a gas chromatograph and a tube furnace whose original purpose aimed to provide basic oxidation data of graphite under accident conditions of a HTGR^24,26^. A mercury porosimeter measured the microstructure of the pristine and oxidized specimens. The higher linearity and smaller standard errors of Arrhenius plots also indicated the applicability and rationality of the proposed method. Future works were discussed concerning a wider range of gas flow rate and O_2_ concentration and more types of graphite.

## Results

### Results of oxidized graphite with related high gas flow rate

**Figure 1:**
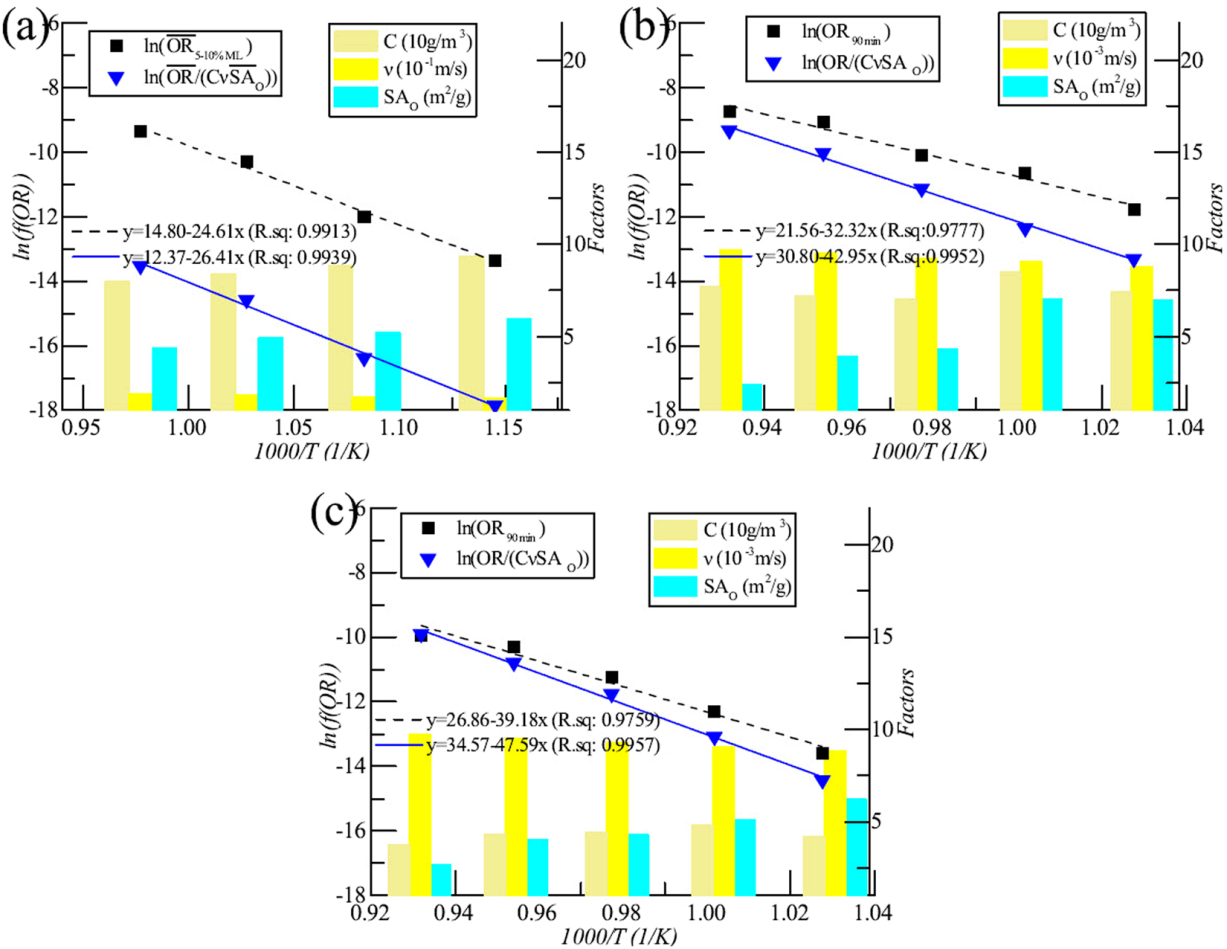
Arrhenius plot of graphite ET-10 and IG-110. (**a**) IG-110, 10 L/min, 21 mol% O_2_; (**b**) ET-10, 0.2 L/min, 20 mol% O_2_; (**c**) ET-10, 0.2 L/min, 10 mol% O_2_.

Oxidation Rate (OR) and its related pore area of graphite IG-110 were obtained from the previous work of Wang *et al*.^32^. The specimens were oxidized based on the experiment conditions (10 L/min air flow) recommended by ASTM D7542 and their pore areas were obtained based on optical microscopy examination. The Arrhenius plots, i.e. the temperature dependence of OR, are shown in Fig. 1(a). The Arrhenius plot labeled with $ln({\overline{OR}}_{5{\textstyle \mbox{--}}10{\rm{ \% }}ML})$ uses the average oxidation rates from 5% ML to 10% ML for calculating the activation energy according to ASTM D7542. The Arrhenius plot labeled with $ln(\overline{OR}/(C\nu {\overline{SA}}_{O}))$ is based on the proposed method by this paper regarding various factors such as O_2_ concentration (C), gas flow rate (*ν*) and surface area of open pore of graphite (SA_*O*_). Here, the values of $\overline{OR}$ and ${\overline{SA}}_{O}$ are also the average values from 5% ML to 10% ML of specimens. The values of various factors at difference temperature are shown as the bar graphs. The gas flow rates and the O_2_ concentrations at different temperature are calculated based on the state equation of idea gas. The open surface areas of the oxidized graphite at different temperature are obtained according Equation (16). The fitting results with R^2^ values are shown in this figure. The results based on our proposed method have better linearity with higher R^2^ values.

Table 1 compares the results obtained by the method recommended by ASTM D7542 and the proposed method. The obtained activation energy of graphite IG-110 (220 kJ/mol) based on the proposed method is closer to those of other studies (218 kJ/mol^12^ and 222 kJ/mol^19^) with sufficient O_2_ supply comparing with 201 kJ/mol^21^ and 205 kJ/mol^32^ based on experiment conditions recommended by ASTM D7542 standard. In addition, the standard errors of the proposed method are smaller. This calculation result is also consistent with the argument that the experiment conditions of ASTM D7542 needed adjustment to provide enough O_2_ for oxidation of graphite IG-110^24^.

**Table 1:** Fitting results using different oxidation rates.

Graphite	C(O_2_) (mol%)	*f*(*OR*)	Ea (kJ/mol)	ln(B)
IG-110	21	${\overline{OR}}_{5-\mathrm{10 \% }ML}$	204.60 ± 13.54	14.80 ± 1.73
IG-110	21	$\overline{OR}/(C\nu {\overline{SA}}_{O})$	219.54 ± 12.16	12.37 ± 1.55
ET-10	20	*OR*_90 *min*_	268.71 ± 23.45	21.56 ± 2.76
ET-10	20	*OR*/(*CνSA*_*O*_)	357.09 ± 14.38	30.80 ± 1.69
ET-10	10	*OR*_90 *min*_	325.74 ± 29.57	26.86 ± 3.48
ET-10	10	*OR*/(*CνSA*_*O*_)	395.69 ± 15.01	34.57 ± 1.77

**Figure 2:**
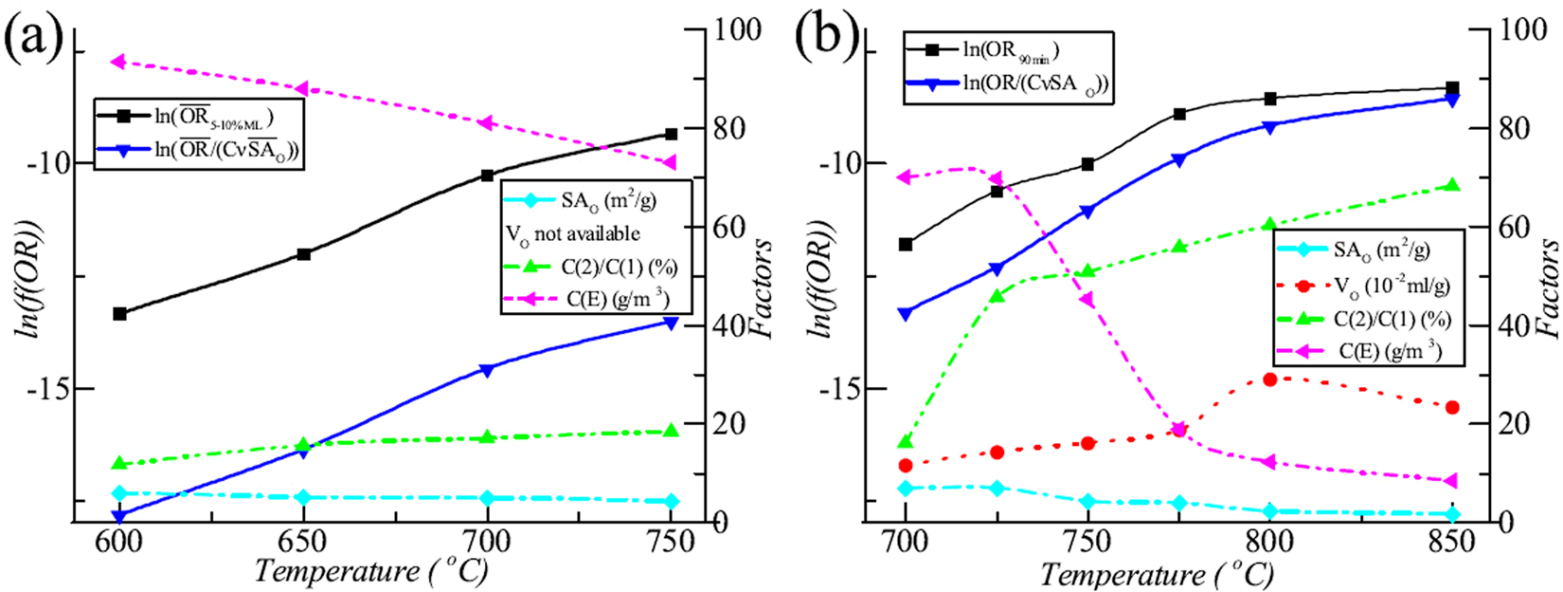
Oxidation factors versus oxidation rates. (**a**) IG-110, 10 L/min, 21 mol% O_2_; (**b**) ET-10, 0.2 L/min, 20 mol% O_2_.

The surface area of open pore positively related with the reaction area between graphite and O_2_ and the volume of open pore positively related with the O_2_ supply and the reaction volume of CO combustion. Graphite oxidation also positively related with CO combustion since graphite oxidation is the origin of CO. Previous study proposed a method to distinguish O_2_ consumed by oxidation reaction and by CO combustion^24^. Here, Fig. 2(a) shows the relations between various factors, such as microstructure of graphite IG-110, O_2_ supply (O_2_ in exhaust (*C*(*E*)) and ratio of O_2_ consumed by CO combustion (*C*(2)) to that by oxidation reaction (*C*(1))) and OR. Here, we include the average ORs from 5% ML to 10% ML and the revised ORs combing O_2_ supply and surface areas of open pore. The $ln(\overline{OR}/(C\nu {\overline{SA}}_{O}))$ had slightly higher linearity than the $ln({\overline{OR}}_{5{\textstyle \mbox{--}}10{\rm{ \% }}ML})$. Since O_2_ was redundant (*C*(*E*) was high) at most temperature (except at 750 °C), ORs were mainly determined by the temperature effect.

### Results of oxidized graphite with low gas flow rate

The nuclear graphite ET-10 was oxidized by the mixture gas (O_2_ and helium) with 0.2 L/min mixture gas considering the air ingress accident of HTGR. The mole fraction of O_2_ was 10 mol% or 20 mol%.

Figure 1(b,c) indicates the Arrhenius plots of the oxidized graphite ET-10 with different O_2_ concentrations. The temperature range is 700–800 °C because the linearity became obviously worse when adopting the data at 850 °C. The Arrhenius plot labeled with *ln*(*OR*_90*min*_) uses the OR when the oxidation time was 90 minutes. The Arrhenius plot labeled with *ln*(*OR*/(*CνSA*_*O*_)) is based on the proposed method regarding various factors such as O_2_ concentration, gas flow rate and surface area of open pore of graphite at the time point of 90 minutes. The values of various factors at difference temperature are shown as the bar graphs. The gas flow rates and the O_2_ concentrations at different temperature are calculated based on the state equation of idea gas. The open surface areas of the oxidized graphite are measured by a mercury porosimeter. The fitting results with R^2^ values are shown in these figures. The results based on proposed method have better linearity with higher R^2^ values.

Table 1 includes the calculation results of graphite ET-10. The standard errors of the proposed method are obvious smaller. In addition, it gets closer pre-exponential and activation energies at different O_2_ concentrations.

Here, Fig. 2(b) shows the relations between various factors, such as microstructure of graphite ET-10, O_2_ supply and OR. The *ln*(*OR*_90*min*_) had obvious worse linearity than the *ln*(*OR*/(*CνSA*_*O*_)). When O_2_ was redundant (*C*(*E*) was high) from 700 °C to 725 °C, rates of oxidation reaction were mainly determined by the temperature effect. The $ln({\overline{OR}}_{90min})$ and $ln(\overline{OR}/(C\nu {\overline{SA}}_{O}))$ had close linearity. At 750–850 °C with the increased shortage of the O_2_ supply (*C*(*E*) became smaller), the influences of microstructure of graphite (the surface area and volume of open pore) and the actual O_2_ supply regarding O_2_ consumed by CO combustion were become stronger. The $ln({\overline{OR}}_{90min})$ had worse linearity than the $ln(\overline{OR}/(C\nu {\overline{SA}}_{O}))$.

**Figure 3:**
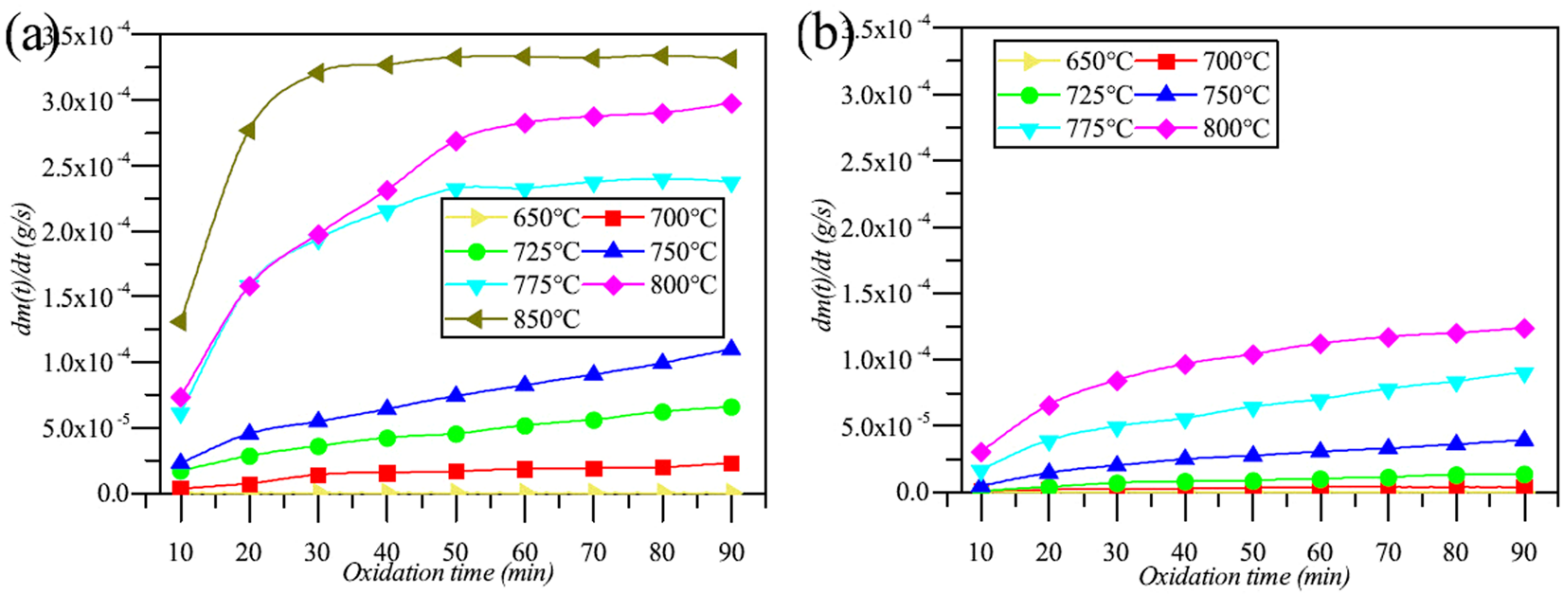
Mass loss rates of specimen. (**a**) ET-10, 20 mol% O_2_; (**b**) ET-10, 10 mol% O_2_.

**Figure 4:**
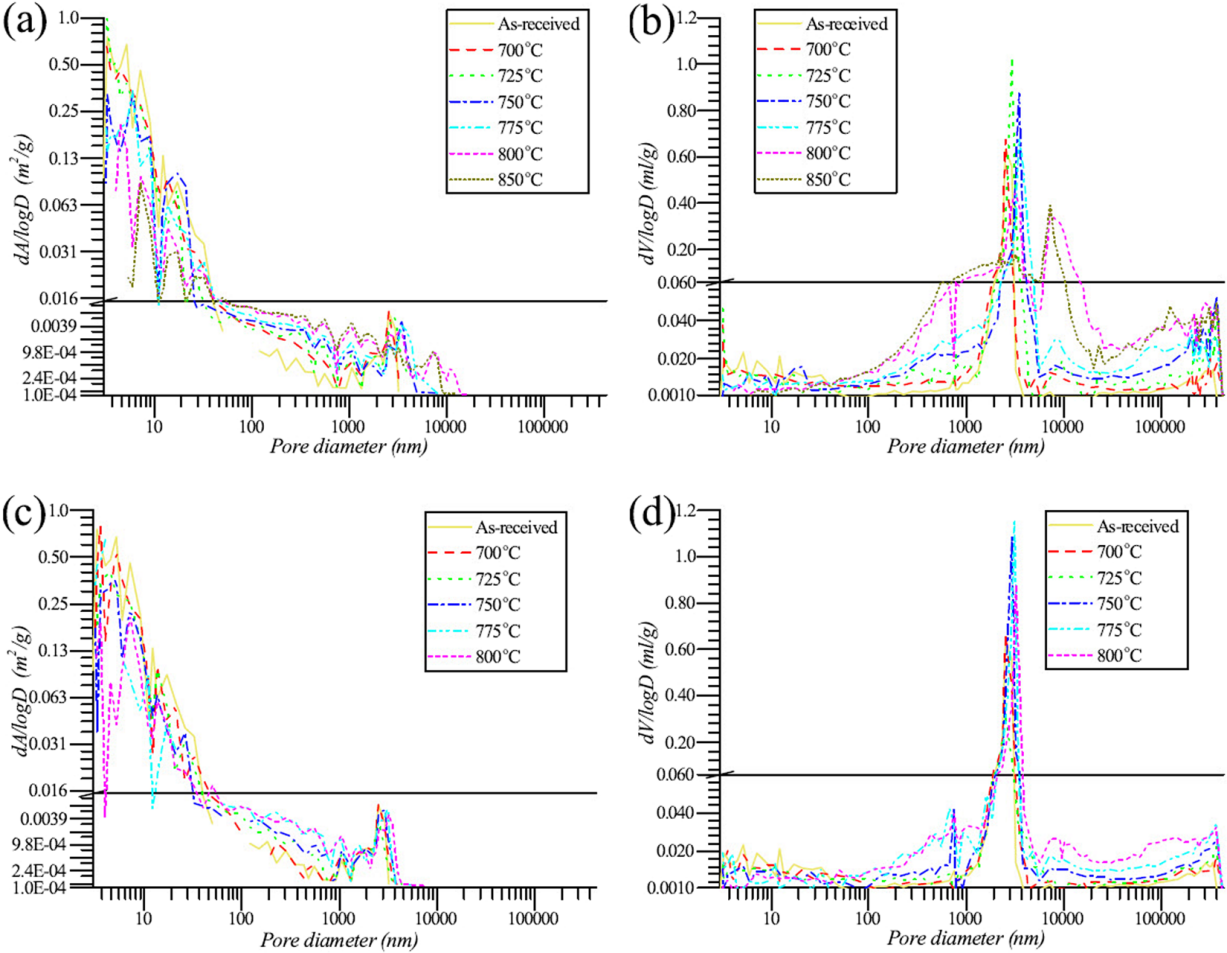
Microstructure of graphite ET-10 oxidized by mixture gas (20 or 10 mol% O_2_) with flow rate of 0.2 L/min for 90 minutes. (**a**) Surface area of open pore, 20 mol% O_2_; (**b**) Volume of open pore, 20 mol% O_2_; (**c**) Surface area of open pore, 10 mol% O_2_; (**d**) Volume of open pore, 10 mol% O_2_.

The ML rates of the specimens are shown in Fig. 3. The ML rates increased with the increase of the oxidation temperature. In addition, the O_2_ concentration had a positive effect on the oxidation rate. Figure 4 shows the microstructure of the specimens oxidized at different temperature for 90 minutes. In general, the surface area and volume of small open pore (diameter < 30 nm) decreased with the increase of temperature. The surface area and volume of middle open pore (30 nm < diameter < 3000 nm) and big open pore (diameter > 3000 nm) increased with the increase of temperature. Totally, the surface area of open pore of graphite ET-10 decreased, and oppositely, the volume increased. An exception is at 850 °C where the volume of big pore was smaller than that at 800 °C resulting in the decrease of volume of open pore from 800 °C to 850 °C.

## Discussion

The present method for characterizing the O_2_ oxidation behaviors mainly concerned the influence of temperature on reaction rate based on ML (from 5% to 10%) of graphite^15,18,31^. The O_2_ supply is predicted to be sufficient if the conditions of ASTM D7542 is obeyed strictly^18^. The microstructure of graphite, such as surface area, can be ignored or considered as a constant object at same ML range (5–10%) which was independent from temperature and O_2_ supply^16,17^. In this way, the ORs are almost stable especially at the same ML range (5–10%) and the average value of it can be used for calculation of activation energy. Related results on graphite NBG-10, PGXW and R4650 also proved it^31^.

**Figure 5:**
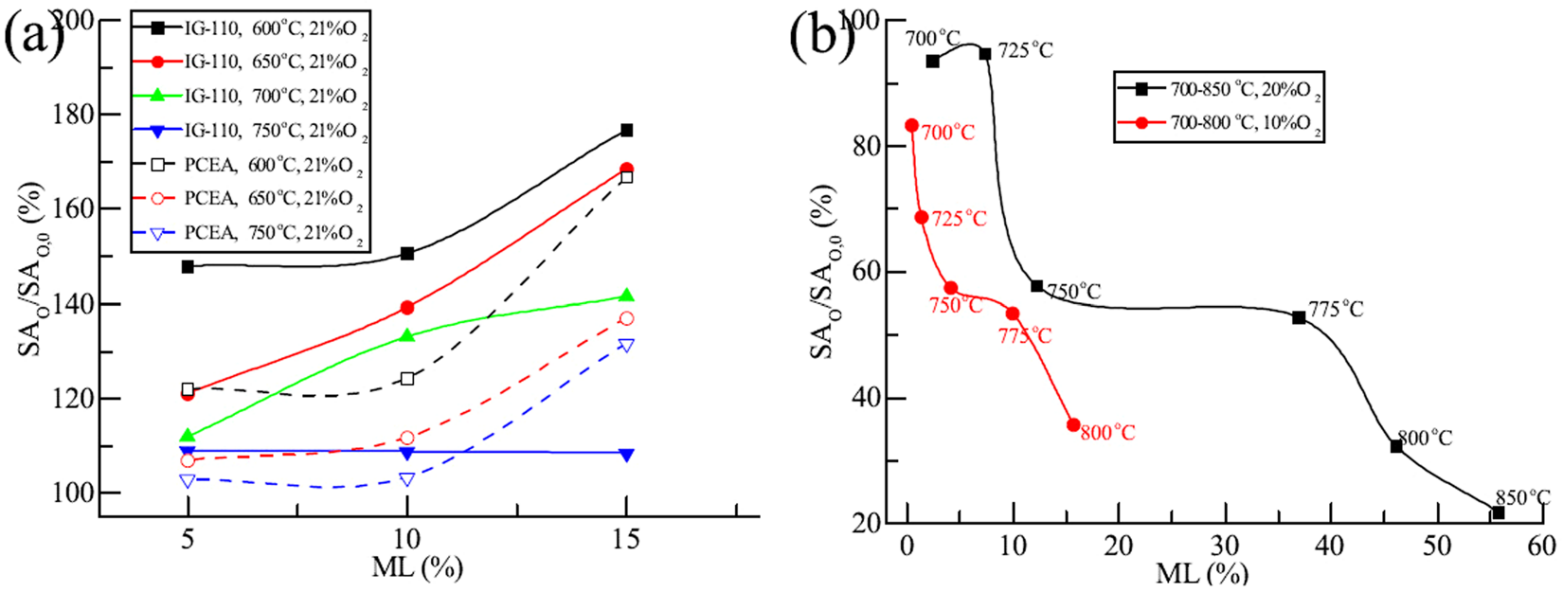
Microstructure of graphite IG-110, PCEA and ET-10 versus mass loss. (**a**) IG-110 and PCEA, 600–750 °C, 21 mol% O_2_; (**b**) ET-10, 700–850 °C, 20 and 10 mol% O_2_.

However, O_2_ oxidation of graphite and CO combustion interact through temperature, graphite supply (microstructure) and O_2_ supply (concentration of O_2_ and flow rate of reactant gas) resulting in quick change of the adequacy of O_2_ supply (ratio of O_2_ supply to consumed O_2_) for some graphite. The microstructure of some graphite oxidized under the ASTM D7542 conditions, such as graphite IG-110, may be quite different even at same ML range (5–10% as required by ASTM D7542) when the oxidation temperature was different^32^ (Fig. 5(a)). According to the experiment results by Contescu *et al*.^21^, the increased of OR was apparently higher than those of other graphite, such as graphite PCEA and NBG-18 under same conditions of ASTM D7542. This indicated the adequacy of O_2_ supply (the ratio of O_2_ supply to consumed O_2_) for oxidation of graphite IG-110 was obviously lower than those of graphite PCEA and NBG-18.

According to Fig. 5(a), the surface area of open pore of graphite IG-110 (real line) decreased more quickly than that of graphite PCEA (dashed line) with the increase of the temperature within 5–10% ML. The adequacy of O_2_ supply indicated by the ratio of O_2_ supply to consumed O_2_ had a direct link with the graphite microstruture, such as surface area of open pore. The more quickly the adequacy of O_2_ supply changes, the more quickly the surface area varies. The more adequate the O_2_ supply is, the higher the surface area is (for same graphite). In addition, the surface areas with different temperature and different ML (within 5–10% ML) became closer with the increased adequacy of O_2_ supply implicating its increased independence from oxidation temperature and O_2_ supply (gas flow rate and O_2_ concentration).

The situation of graphite PCEA can be indicated as the sufficient O_2_ supply since the surface areas are almost independent from ML, temperature and O_2_ supply within 5–10% ML, same as that mentioned by El-Genk and Tournier^16,17^. Of course, the absolute independence is impossible for any actual graphite. On the contrast, the situation of graphite IG-110 is far away from the sufficient O_2_ supply where it is only sufficient at 600 °C within 5–10% ML and nearly sufficient at 650 and 700 °C at 10% ML. Because the above complexities of adequacy of O_2_ of graphite IG-110, the change of oxidation conditions close to ASTM D7542 will apparently change the adequacy of O_2_ supply. And therefore, the interaction between the changes of the adequacy of O_2_ supply and the graphite microstructure results in variations of behavior of ORs with temperature, and finally different values of activation energy. This is the main reason why the calculation results of activation energy were quite different^12,19–22,32^ for graphite IG-110. Among them, the more adequate O_2_ supply increased the surface area of oxidized graphite and the OR at high temperature finally resulting in higher calculated values of activation energy (218 kJ/mol^12^ and 222 kJ/mol^19^).

Our proposed method, namely calculating activation energy considering microstructure of graphite, can mediate the influences of O_2_ supply on microstructure of graphite, OR and activation energy, and therefore it gets closer results as those with more adequate O_2_ supply. Figure 1(a) and Table 1 showed the higher linearity and the lower standard error of the fitting based on our method.

When observing the microstructure of oxidized graphite ET-10 with much lower O_2_ supply, 0.2 L/min reactant gas, the micro surface areas of oxidized graphite ET-10 were usually less than that of pristine graphite (Fig. 5(b)). In general, the surface area of open pore decreased with the increase of ML and temperature. The Arrhenius plot combining the surface area of open pore and O_2_ supply was improved with less standard error (Table 1) and higher linearity (Fig. 5(b)). In addition, calculation results became more reasonable with closer pre-exponential factors and activation energies at different O_2_ concentrations.

Our proposed method is applicable for not only high gas flow (10 L/min) but also related low gas flow (e.g. 0.2 L/min) regarding the situation of air ingress accident. It can be easily conducted by getting the OR at the end of experiment and measuring the microstructure of the oxidized graphite. At present, the mercury porosimeter is recommended for measuring the microstructure since it can obtain the information at a proper range of open pore (diameter from 3 nm to 400,000 nm). The experiment facilities in other studies can be easily shifted to our proposed method. The time of the oxidation experiment can be independent from the ML of graphite which should be longer than 40 minutes (60 minutes is better) to avoid the beginning stage of graphite oxidation with possible rapid rate change.

For the method characterizing the graphite oxidation at high gas flow rate, the recommend experiment conditions by ASTM D7542, such as air flow rate or/and geometry of specimen, should be adjusted to provide more adequate O_2_ to oxidize some popular graphite, such as graphite IG-110, especially at related high temperature. In this way, we can calculated activation energy based on ML, OR and temperature because the influence of microstructure of graphite is predicted to be small.

For the method characterizing the graphite oxidation at low gas flow rate, the experiment and calculating method should take account into both O_2_ supply and microstructure of oxidized graphite. The microstructure of graphite, especially the surface area of open pore, should be provided together with the activation energy.

**Figure 6:**
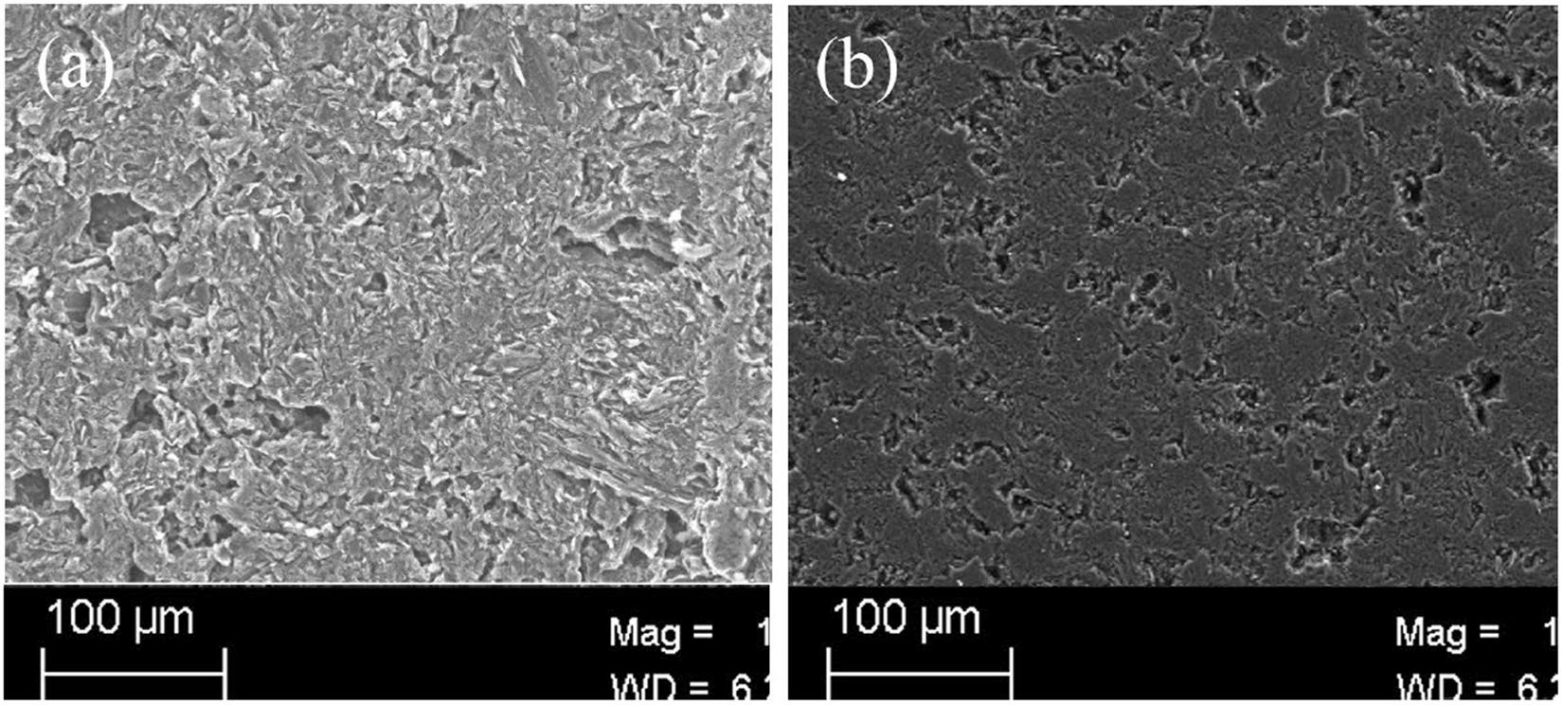
SEM pictures of surface of pristine graphite IG-110 and ET-10. (**a**) IG-110; (**b**) ET-10.

The activation energy of nuclear graphite ET-10 obtained by our study, 357 kJ/mol (20 mol% O_2_) or 396 kJ/mol (10 mol% O_2_), is much higher than those of other graphite, usually around 200 kJ/mol. Although the comparison of them is not our purpose, some explanations may be needed. One reason is because the nuclear graphite ET-10 for HTGR is a newly developed graphite which is not produced in a commercial scale at present. The main properties and main impurities of graphite ET-10 and IG-110 are shown in Tables 2 and 3 respectively. The quality of obtained specimens of graphite ET-10, such as impurity, is predicted to be lower than that provided by the manufacture (Table 3) which is predicted as the upper limit of future products for the commercial scale. The metallic impurities, such as V, K, Fe, Ca, Al and Mg, usually have a catalytic effect on graphite oxidation by reducing the activation energy^33^. The contents of K and V in graphite ET-10 is apparently lower than those in graphite IG-110. Among the main metallic purities in Table 3, K and V are the strongest accelerators on the OR of graphite^34^. The second reason may exist in the surface of the test specimen. The SEM pictures of surface of pristine graphite IG-110 and ET-10, Fig. 6, shows the smaller number of powder and defect of specimen ET-10. The test specimens of nuclear graphite ET-10 were provided piece by piece by the manufacturer. On contrast, other graphite was usually provided in a big block by the manufacturer and then was machined to small specimens by the experimenters. The powder or defect may accelerate the oxidation of graphite especially at related low temperature. The recent study indicated that the ignition temperature of the specimen of graphite IG-110 machined by experimenters is around 400 °C with 0.2 L/min reactant gas flow while the ignition temperature of graphite ET-10 machined by the manufacturer is around 700 °C with same oxidation conditions^24^. The third reason is due to the calculation method. Our proposed method usually got higher values of activation energy because of the decreased surface area of oxidized graphite with the increase of temperature. The fourth reason is the different temperature ranges, 700–800 °C for graphite ET-10 and 600–750 °C for graphite IG-110. The activation energy sometimes depended on the temperature range at which the graphite was oxidized.

**Table 2:** Main properties of graphite ET-10 and IG-110.

Item	ET-10	IG-110^35^
Source coke	Coal tar pitch	Petroleum
Forming process	Isostatic molding	Isostatic molding
Impregnation	None	Have
Bulk density (g/cm^3^)	1.75	1.78
Grain size (*μ*m)	∼15: fine-grain	∼20: fine-grain
Compressive Strength (MPa)	98	76.8
Thermal Conductivity (W/(m·K))	104.4	80
Coefficient of thermal expansion (10^−6^/°C)	3.8	4.06
Young’s modulus (GPa)	10.8	7.9
Open porosity (%)	15	18.39^36^
Impurities (ppm)	<20	<20^12^

**Table 3:** Main impurities of graphite ET-10 and IG-110.

Impurities	ET-10	IG-110^19^
B (ppm)	<0.1	0.15
Si (ppm)	<1.0	0.15
Ca (ppm)	—	0.08
Fe (ppm)	<1.2	0.06
Al (ppm)	<0.1	0.012
K (ppm)	—	0.04
V (ppm)	—	0.018
Mg (ppm)	<0.1	—

—not detected.

Influences of CO combustion and the volume of open pore on oxidation rate increased with the increase of temperature and the decrease of gas flow rate. When characterizing the graphite oxidation, we may need further consideration of CO combustion and volume of open pore especially at related high temperature and low gas flow rate. CO combustion can change not only the contents in the exhaust gas and the actual O_2_ supply to graphite oxidation but also energy balance of graphite oxidation because of its related high reaction heat.

We are now also planning the oxidation experiments of graphite ET-10 under conditions close to ASTM D7542 and the oxidation experiments of graphite IG-110 at low gas flow rates. The microstructure of oxidized graphite will be measured by a mercury porosimeter and other means. Further studies also include related experiments on other graphite with different grain sizes and porosities, such as PCEA, NBG-18 and NBG-25.

## Methods

### Method for calculating activation energy

Fundamentally, the reaction rate of O_2_ oxidation of graphite at a time point (*t*) relates with oxidation temperature (*T*), activation energy of graphite oxidation (*E*_*a*_) and reactant supply including gas flow rate (*ν*), O_2_ concentration (*C*) and graphite microstructure (*MS*):

3 $$OR(t,\,T(t))=f(T(t),\,{E}_{a},\,\nu (t,\,T(t)),\,C(t,\,T(t)),\,MS(t,\,T,\,\nu ,\,C))$$

According to definition of activation energy, we get:

4 $$OR(t,\,T)=f(\nu (t,\,T(t)),\,C(t,\,T(t)),\,MS(t,\,T,\,\nu ,\,C)){e}^{-\frac{{E}_{a}}{RT(t)}}$$

Usually, the surface area of open pore of graphite is the main factor of microstructure related with graphite oxidation. If the reaction temperature and O_2_ supply (gas flow rate and mole fraction of O_2_) at ordinary condition are stable, then we can get:

5 $$OR(t,\,T)=f(\nu (T),\,C(T),\,S{A}_{O}(t,\,T,\,\nu ,\,C)){e}^{-\frac{{E}_{a}}{RT}}$$

In case of oxidation using air with same volume flow rate such as 10 L/min, the mole fraction of O_2_ is around 21% and the values of *C* at different temperature in kinetic regime are close. In addition, the values of *ASA* (Active Surface Area) at different temperature in kinetic regime were usually determined by the ML of graphite independent from the oxidation conditions such as temperature, O_2_ concentration and gas flow rate^16,17^:

6 $$OR({t}_{ML},\,T)=f(\nu ,\,C,\,ASA({t}_{ML})){e}^{-\frac{{E}_{a}}{RT}}$$

If the experiments for different oxidation temperature measured the average value of oxidation rate at same Mass Loss Range (*MLR*), the average value of ASA will be a nearly constant value. In other words, the oxidation rate will be nearly constant in the period during which the change of ML is small. The experiment study for some graphite (PGXW, NBG-10 and R4-650) confirmed this situation where the oxidation rate of related graphite became nearly constant when the ML was from 5% to 10%^31^. In this way, we get:

7 $${\overline{OR}}_{MLR}(T)=f(\nu ,\,C,\,AS{A}_{MLR}){e}^{-\frac{{E}_{a}}{RT}}=Z{e}^{-\frac{{E}_{a}}{RT}}$$

where *Z* is a constant pre-exponential factor.

Finally, the activation energy of graphite in kinetic regime can be calculated according to the slop of Arrhenius plot based on the recommended condition by ASTM D7542^15^:

8 $$ln({\overline{OR}}_{MLR}(T)=ln(Z)-\frac{{E}_{a}}{RT}$$

However, several studies^12,19–22^ found the activation energies of graphite IG-110 were quite conditionally dependent on O_2_ supply even if the experiment condition was close to that recommended by ASTM D7542. The change of pore areas of oxidized graphite IG-110 is determined not only by ML but also by oxidation temperature and possibly other factors, such as oxidant flow rate and O_2_ concentration. Graphite IG-110 demonstrated the obvious decrease of surface area with the increase of oxidation temperature (600, 650, 700 and 750 °C) at same ML (5% and 10%)^32^ which was different from the predicted stableness of surface areas^16,17^.

In addition, regarding the actual situation of HTGR, some other studies^19,26–28^ had to concern the oxidation behaviors of non-standard shape graphite with a much lower gas flow. The rationality of the results of these studies was in doubt since the calculation method and experiment conditions recommended by ASTM D7542 were required to be strictly obeyed^18^.

Furthermore, even at same MLR of 5–10%, some recent studies revealed that the complexities of ORs when increasing the oxidant flow rate^25,26^. These phenomena suggested the influence of flow rate on micro surface area cannot be ignored for some graphite.

In summary, the influence of gas flow rate, O_2_ concentration and microstructure of some graphite on OR under different temperature cannot be combined to a constant pre-exponent factor:

9 $$OR(t,\,T)=f(\nu (t,\,T(t)),\,C(t,\,T(t)),\,MS(t,\,T,\,\nu ,\,C){e}^{-\frac{{E}_{a}}{RT(t)}}\ne Z{e}^{-\frac{{E}_{a}}{RT}}$$

Consequently, when characterizing the kinetic parameters of graphite, we need to consider the changes of microstruture, O_2_ concentration and oxidant flow rate. If the contributions of graphite microstruture, O_2_ concentration and oxidant gas flow rate are considered equally and surface area of open pore is applied to represent the microstruture, then:

10 $$OR(t,\,T)=B\nu (t,\,T)C(t,\,T)S{A}_{O}(t,\,T,\,\nu ,\,C){e}^{-\frac{{E}_{a}}{RT}},$$

where, B is a pre-exponential factor.

We can calculate the apparent activation energy of graphite according to linearized form of the following equation:

11 $$ln(\frac{OR(t,\,T)}{\nu (t,\,T)C(t,\,T)S{A}_{O}(t,\,T\,,\,\nu ,\,C)})=ln(B)-\frac{{E}_{a}}{RT}$$

where the units of *OR*, *ν*, *C* and *SA*_*O*_ are g/(g ⋅ s), m/s, g/m^3^ and m^2^/g respectively for actual calculation.

Here, the OR can be indicated by:

12 $$OR(t,\,T)=\frac{dm(t,\,T)}{dt}\frac{1}{m(t,\,T)}$$

where *m* is residual mass of specimen.

The ML rate of the graphite is calculated according to the contents of CO_2_ and CO in the exhaust gas:

13 $$\frac{dm(t,\,T)}{dt}=\frac{{\nu }_{v}(t,\,T)(f(C{O}_{2}(t,\,T))+f(CO(t,\,T))){\rho }_{a}{M}_{c}}{{M}_{a}}$$

where *t* is oxidation time in s, *m* is residual mass of specimen in g, *ν*_*v*_ is the volume flow rate of in *m*^3^/s, *f*(*C*O_2_) and *f*(*CO*) are mole fractions of CO_2_ and CO in exhaust gas respectively, *ρ*_*a*_ is density of air at ordinary temperature and pressure with value of 1.293 g/m^3^, M _*c*_ is atomic weight of carbon element and M _*a*_ is average molecular weight of air.

For the studies regarding the graphite oxidation under accident conditions of nuclear reactor, the time after the air ingress accident were usually concerned and therefore these studies usually adopted the OR according to the time points after the beginning of oxidation, such as 1 or 3 hours^11^, 60 or 80 minutes^24,26^ and 4 hours^27^. Here, we also included calculation result based on the OR at the same time point, 90 minutes after the beginning of oxidation. The activation energy can be calculated by:

14 $$ln(O{R}_{t})=ln(Z)-\frac{{E}_{a}}{RT}$$

### Test specimen and conditions

The test specimen of nuclear graphite ET-10 was provided by IBIDEN Co. Ltd., Japan. The main properties of graphite ET-10 are shown in Table 2. The main impurities of graphite ET-10 are shown in Table 3 which are provided by the manufacturer. The dimensions of the oblate rectangular specimen are 30.0 mm × 29.5 mm × 1.95 mm. The graphite ET-10 was oxidized by the 0.2 L/min oxidant gas (O_2_ (10 or 20 mol%) and helium) at 650–850 °C.

The test specimen of nuclear graphite IG-110 was provided by Toyo Tanso Co. Ltd., Japan. The main properties of Graphite IG-1110 are also shown in Table 2. The main impurities of graphite IG-110 are shown in Table 3. The experiment conditions^32^ are same as those recommended by ASTM D7542^15^. The cylinder specimen with a 25.4 mm diameter and a 25.4 mm length were oxidized by the 10 L/min air flow at 600–750 °C.

### Calculating micro surface area of oxidized graphite IG-110

The microstructure of the oxidized graphite IG-110 based on the experiment conditions (10 L/min air flow) recommended by ASTM D7542 was measured based on optical microscopy xamination^32^. Although the values of the Pore Area (PA) obtained by a optical microscopy on a cross section were far away from the values obtained by a mercury porosimeter in this paper and another study^25^, it provided quantitative variations of oxidized graphite IG-110 at different temperature. Since the surface area of open pore of pristine graphite IG-110 was around 4 m^2^/g^25^, we used a direct scale relation to calculate the surface area of the oxidized graphite IG-110 according to the surface area obtained by the previous study^32^:

15 $$\frac{S{A}_{O}}{S{A}_{O\mathrm{,0}}}=\frac{PA}{P{A}_{0}}$$

where subscript _0_ indicates the related value of the pristine graphite.

Because the change of the surface area with temperature, not the absolute values of it, determines the calculation of activation energy, this conversion did not change the calculation result of activation energy. It only made the situation more comparable, e.g. that in Fig. 1.

### Test facility and procedure

The test facility for oxidizing the graphite ET-10 and measuring components of the exhaust gas is same as the previous study^26^. O_2_ and helium flowing through related mass flow meters are mixed in a mixer, and the pressure of mixed gas is reduced by a pressure reducing valve before entering a quartz reaction tube heated by three electric heaters. The quartz tube has a 40 mm diameter and a 1200 mm length. The heating area is divided into three zones heated by three electric heaters separately whose temperature is detected by three Pt-Rh thermocouples respectively. The specimen lying on a ceramic crucible is located in the middle heating zone. In the quartz tube, another thermocouple is inserted to the side of the specimen to detect the temperature of specimen.

The components of the exhaust gas produced by oxidation reaction are measured by an on-line gas chromatography (GC-1100, Beijing PERSEE General Instrument, INC.) after flowing through a counterbalance valve. The surface areas and volumes of the open pore of the pristine and oxidized specimens are measured by a mercury porosimeter (AutoPore IV 9500, Micromeritics Instrument Corp.).

Before the oxidation reaction, pure helium (99.995%) was injected into the quartz tube. Then the test specimen was heated to a certain temperature in the inert atmosphere whose process took around 90 minutes. After that, pure O_2_ (99.999%) and pure helium were mixed and injected into the quartz tube to oxidize the graphite for 90 minutes. At the same time, the contents of exhaust gas were measured by the gas chromatography. Finally, the test specimen was cooled to the room temperature in an inert atmosphere.

## Data Availability

The datasets generated during and/or analysed during the current study are available from the corresponding author on reasonable request.
